# Preoperative Magnetic Resonance Cholangiopancreatography for Detecting Difficult Laparoscopic Cholecystectomy in Acute Cholecystitis

**DOI:** 10.3390/diagnostics11030383

**Published:** 2021-02-24

**Authors:** Kojiro Omiya, Kazuhiro Hiramatsu, Yoshihisa Shibata, Masahide Fukaya, Masahiro Fujii, Taro Aoba, Atsuki Arimoto, Takayuki Yamaguchi, Takehito Kato

**Affiliations:** Department of General Surgery, Toyohashi Municipal Hospital, 50 Hakken-Nishi, Aotake-Cho, Toyohashi City, Aichi Prefecture 440-8570, Japan; hiramatsu-kazuhiro@toyohashi-mh.jp (K.H.); shibata-yoshihisa@toyohashi-mh.jp (Y.S.); fukaya-masahide@toyohashi-mh.jp (M.F.); fujii-masahiro@toyohashi-mh.jp (M.F.); aoba-taro@toyohashi-mh.jp (T.A.); arimoto-atsuki@toyohashi-mh.jp (A.A.); yamaguchi-takayuki@toyohashi-mh.jp (T.Y.); kato-takehito@toyohashi-mh.jp (T.K.)

**Keywords:** acute cholecystitis, cholecystectomy, MRCP, MRI

## Abstract

Previous studies have shown that signal intensity variations in the gallbladder wall on magnetic resonance imaging (MRI) are associated with necrosis and fibrosis in the gallbladder of acute cholecystitis (AC). However, the association between MRI findings and operative outcomes remains unclear. We retrospectively identified 321 patients who underwent preoperative magnetic resonance cholangiopancreatography (MRCP) and early laparoscopic cholecystectomy (LC) for AC. Based on the gallbladder wall signal intensity on MRI, these patients were divided into high signal intensity (HSI), intermediate signal intensity (ISI), and low signal intensity (LSI) groups. Comparisons of bailout procedure rates (open conversion and laparoscopic subtotal cholecystectomy) and operating times were performed. The recorded bailout procedure rates were 6.8% (7/103 cases), 26.7% (31/116 cases), and 40.2% (41/102 cases), and the median operating times were 95, 110, and 138 minutes in the HSI, ISI, and LSI groups, respectively (both *p* < 0.001). During the multivariate analysis, the LSI of the gallbladder wall was an independent predictor of both the bailout procedure (odds ratio [OR] 5.30; 95% CI 2.11–13.30; *p* < 0.001) and prolonged surgery (≥144 min) (OR 6.10, 95% CI 2.74–13.60, *p* < 0.001). Preoperative MRCP/MRI assessment could be a novel method for predicting surgical difficulty during LC for AC.

## 1. Introduction

Acute cholecystitis (AC) is one of the most common surgical emergencies in the world. The Tokyo Guidelines propose that the treatment strategy should be considered and chosen according to the severity. Early laparoscopic cholecystectomy (LC) soon after onset is recommended as the first choice of treatment for patients with grade I (mild) and grade II (moderate) AC and for selected patients with grade III (severe) AC [1]. However, severe intraoperative complications such as bile duct injury occur at a certain rate in LC [2,3]. Given the number of daily surgeries performed, preoperative assessment to prevent intraoperative complications in each case is crucial. Surgical difficulty due to severe inflammation and an anatomical anomaly of the bile duct, such as an aberrant posterior sectoral hepatic duct (PHD), are the most common causes of serious complications, such as bile duct injury, during LC [2,4,5,6]. In addition, 7.7–14.3% of patients with AC have concomitant common bile duct stones, which need to be treated with 1-stage or 2-stage treatment management [7,8,9]. To determine the appropriate treatment strategy and perform subsequent early LC safely, the surgeon needs the ability to predict the surgical difficulty of LC and assess the biliary anatomy in a limited amount of time before surgery, especially in emergency conditions. 

Magnetic resonance cholangiopancreatography (MRCP) is now widely used to noninvasively assess the biliary tract anatomy without a contrast agent. MRCP is useful for diagnosing biliary disease and its cause, such as common bile duct stones. Additionally, a recent retrospective study reported that MRCP effectively assessed the aberrant PHD, which is frequently injured during LC [10]. Meanwhile, Jung et al. [11] have highlighted that half-Fourier acquisition single-shot turbo spin-echo (HASTE) magnetic resonance imaging (MRI) of AC captured during MRCP typically reveals two layers in the gallbladder wall: A low signal inner layer of mucosa and muscle and a high signal outer layer of serosal edema. Our previous study revealed that the thickening of the inner layer with a low signal was associated with significant inflammatory pathological changes in the gallbladder wall, such as necrosis and fibrosis, which substantially impact the surgical difficulty of LC [12]. Thus far, using rates of bailout procedures (such as open conversion and laparoscopic subtotal cholecystectomy) and the operating time as indicators, numerous studies have identified predictors for surgical difficulty during early LC for AC. However, the relationship between MRI findings and the surgical outcome of early LC for AC has not been clarified. If a surgical difficulty prediction method using HASTE MRI were to be established, MRCP might become the best evaluation method for the comprehensive surgical management of AC and decision making when there is limited time before surgery.

The present study investigated the association between signal intensity variations in gallbladder walls on MRI and surgical outcomes in patients who underwent early LC for AC to elucidate the utility of MRCP for predicting surgical difficulty.

## 2. Materials and Methods

This retrospective single-institution study was performed to discern the association between preoperative MRI findings and surgical outcomes of early LC in patients with AC at Toyohashi Municipal Hospital in Japan between January 2010 and December 2019. The ethics committee of Toyohashi Municipal Hospital approved the study protocol (approval number 569).

### 2.1. Patients

We searched an institutional surgery database to identify eligible patients, and we collected patient information from the electronic medical records. The eligibility criteria were as follows: (1) AC clinically diagnosed according to the Tokyo Guidelines of 2007 [13], 2013 [14], and 2018 [15]; (2) having undergone LC ≤ 7 days from disease onset (early LC); and (3) having undergone MRCP/MRI ≤ 24 h before surgery. Study exclusion criteria included (1) a gallbladder wall thickness of less than 3 mm on MRI and (2) the clinical suspicion of gallbladder cancer. 

During the study period, computed tomography (CT) was performed for all patients clinically suspected of having AC, for the differential diagnosis and an assessment of the general condition. Surgical indications for AC and types of surgery followed the Tokyo Guidelines in principle, but were ultimately decided on a case-by-case basis by the surgeon. Most of the patients who presented at 72 h or later after onset underwent elective surgery according to the 2013 Tokyo Guidelines [16]. Preoperative MRCP on a 3-T superconducting instrument (MAGNETOM Skyra; Siemens, Erlangen, Germany) was routinely performed on patients diagnosed with AC who were scheduled to undergo early LC to assess the presence of common bile duct stones and abnormal anatomical variations in the bile duct before surgery. If the Calot’s triangle anatomy was unclear due to severe inflammation in intraoperative findings, bailout procedures such as open conversion or laparoscopic subtotal cholecystectomy were considered. The details of each surgery, such as the type of surgery, intraoperative biliary injury, intraoperative accidental injury of the gallbladder, and degree of inflammation as intraoperatively determined by the surgeon, were routinely recorded in the surgical record based on a questionnaire that was administered as soon as the surgery was completed.

### 2.2. MRI Assessment

**Figure 1:**
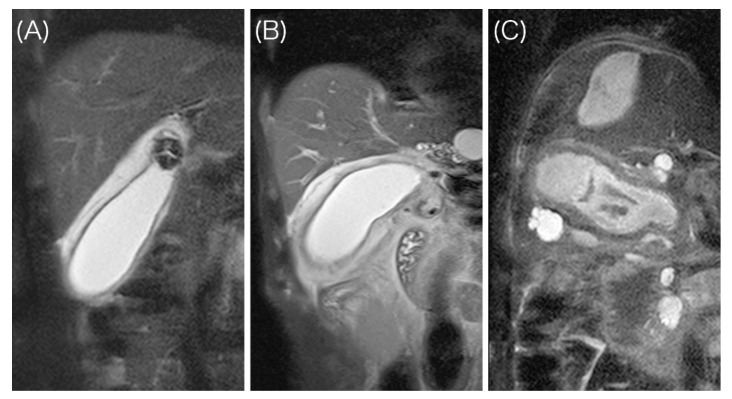
Layered pattern of the gallbladder wall. (**A**) High signal intensity (HSI) type: Thin inner layer with a low signal intensity, thickened outer layer with a high signal intensity, and well-defined margin; (**B**) intermediate signal intensity (ISI) type: Partially thickened inner layer, intermediate signal intensity in the outer layer, and ill-defined margin; (**C**) low signal intensity (LSI) type: Diffusely thickened low signal intensity layer.

HASTE T2-weighted MRI scans were captured during MRCP for all eligible patients and were retrospectively and independently assessed by two surgeons (K. O. and K. H.), who were blinded to the clinical information and type of surgery, but were aware that cholecystitis was present in each case. The gallbladder wall thickness was measured from the section showing the thickest part of the wall. Based on the layered pattern of the thickened wall, patients were divided into three groups as follows (Figure 1):

1. A high signal intensity (HSI) group having two layers with a discrete margin composed of a thin inner layer (≤3 mm) with a low signal and a relatively thick outer layer with a high signal;
2. An intermediate signal intensity (ISI) group having two layers with a partially ill-defined margin composed of a partially thickened inner layer (>3 mm) with a low signal and an outer layer with a high or partially heterogeneous intermediate signal;
3. A low signal intensity (LSI) group having ill-defined layers composed of a diffusely thickened inner layer (>3 mm) with a low signal and an outer layer with an intermediate to low signal.

This classification scheme was the same as that used in our previous study [12]. We determined the signal intensities by adopting standardized regions of interest. The size of the region of interest was similar for all measurements and patients and varied between 0.03 and 0.06 cm^2^. We judged LSI lesions of the gallbladder wall relative to the renal parenchyma’s signal intensity level. 

### 2.3. Outcomes 

#### 2.3.1. Primary Outcomes

The primary outcomes of the study were the ‘’bailout procedures’’ rate, defined as open conversion of the initial laparoscopic approach and laparoscopic subtotal cholecystectomy, and the operating time. Laparoscopic subtotal cholecystectomy was defined as gallbladder resection with a laparoscopic linear stapler at the gallbladder neck. The outcomes among the three groups were compared. 

#### 2.3.2. Secondary Outcomes

Secondary outcomes of the study were blood loss, intraoperative biliary injury, intraoperative accidental injury to the gallbladder, a postoperative hospital stay, reoperation, readmission, and the incidence rate of postoperative complications. Clavien–Dindo scoring was used to grade the postoperative complications. The overall postoperative complication rate was defined as that occurring in grades II to V, and major postoperative complications were defined as grades III to V. Additionally, we reviewed intraoperative finding records on the degree of inflammation, as evaluated by the surgeon. The outcomes among the three groups were compared. 

### 2.4. Identification of Predictors for Increased Surgical Difficulty

We assessed the layered pattern of the gallbladder wall on MRI and other factors potentially predictive of surgical difficulty that had previously been reported [17,18,19,20,21,22,23,24,25,26,27] to identify predictive factors for the bailout procedures and prolonged operating times. These included a male sex, age, the body mass index (BMI), the body temperature, diabetes, gallbladder wall thickening, incarcerated stones in the gallbladder neck, fluid retention around the gallbladder, the white blood cell (WBC) count, the C-reactive protein (CRP) level, the albumin level, the total bilirubin level, the severity grade according to the Tokyo Guidelines [13,14,15], and the time between disease onset and surgery. A prolonged operating time was defined as the third quartile of the operating time of the entire study group. Severe inflammation findings detected on CT were also compared, such as irregular thickening of the gallbladder wall, poor contrast enhancement of the gallbladder wall, an increased density of fatty tissue around the gallbladder, membranous structures within the lumen, and an abscess around the gallbladder. These CT findings were reported as indicators of gangrenous cholecystitis in the study by Bennett et al. [28]. In addition, we assessed the surgeon’s previous experience because some studies have suggested that the operating time greatly varies, depending on the operator’s skill and experience [29,30,31]. 

### 2.5. Statistical Analysis

Continuous variables are presented as medians and interquartile ranges, whereas categorical variables are presented as numbers of patients and percentages. All *p*-values are two-sided, and associations were considered significant at *p* < 0.05. Differences in categorical variables were tested using Fisher’s exact test. The Kruskal–Wallis rank-sum test was used to detect differences in continuous variables among the three signal intensity groups. If there was a significant difference between the three groups, pairwise comparisons for all groups were performed, with *p*-values adjusted using the Holm method.

A univariate analysis with independent variables was performed to identify independent risk factors for bailout procedures and prolonged operating times. Continuous variables were dichotomized based on institutional reference values or the first or third quartile of the entire study group. Those variables with *p* ≤ 0.1 in the univariate analysis were entered into multivariable logistic regression models. The discrimination power of the logistic regression model was summarized using the C-index.

All statistical analyses were performed using R version 3.5.2 (R Foundation for Statistical Computing, Vienna, Austria) and EZR (Saitama Medical Center, Jichi Medical University, Saitama, Japan), the latter of which is a graphical user interface for R. 

## 3. Results

### 3.1. Patient Selection and MRI Assessment

**Figure 2:**
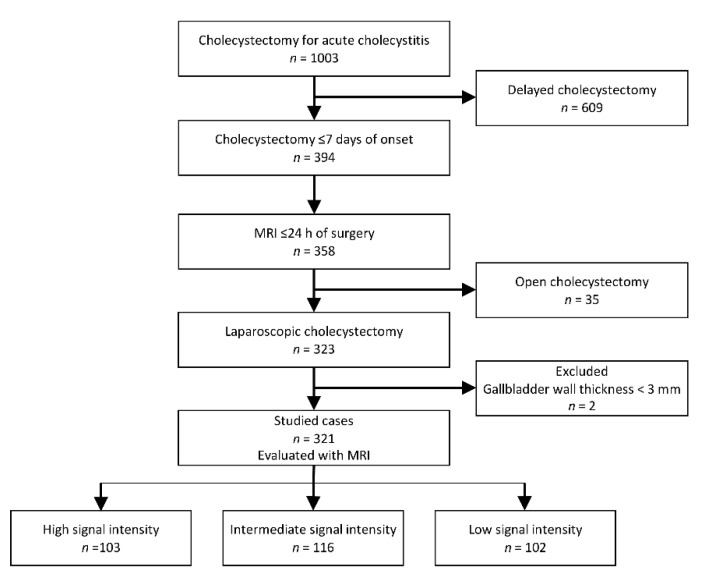
Study population diagram.

Among 1003 patients who underwent cholecystectomy after a diagnosis of AC, 394 underwent early cholecystectomy and 609 underwent delayed cholecystectomy. MRCP detected concomitant common bile duct stones (CBDS) in 160 (16.0%) of 1003 patients, and all of them received two-stage treatment management and underwent delayed surgery. According to the 2013 Tokyo Guidelines recommendations [16], 130 of 159 patients who had been diagnosed with AC more than 72 h after onset underwent delayed surgery. Other reasons for a delay in surgery included severe comorbidities, cholangitis, pancreatitis, and a refusal to undergo early surgery. Of the 394 patients who underwent early cholecystectomy, 358 (90.1%) had undergone preoperative MRI. While 323 patients underwent LC, 35 patients underwent planned open cholecystectomy by the surgeon’s decision due to a history of previous abdominal surgery or radiological findings that indicated severe inflammation of the gallbladder. Two of 323 patients were excluded due to a gallbladder wall thickness of less than 3 mm on MRI. Ultimately, 321 patients were deemed eligible for inclusion. Following the MRI findings assessment, 103, 116, and 102 patients were included in the HSI, ISI, and LSI groups, respectively (Figure 2). Of 35 excluded patients with planned open surgery, 3, 14, and 18 patients showed HSI, ISI, and LSI on MRI, respectively.

PHD anatomies were confirmed on MRCP in 315 (98.1%) of 321 studied patients. Aberrant PHDs were detected in 23 (7.2%) patients.

### 3.2. Patient Characteristics in Each MRI Group

The baseline patient characteristics and preoperative findings for the study population are shown in Table 1; there were no significant differences in patient characteristics. The rate of patients with severe inflammation findings on CT were significantly higher in the ISI (0.0%) and LSI (8.6%) groups than in the HSI (17.6%) group based on a pairwise comparison (*p* = 0.004 (HSI vs. ISI), *p* = 0.067 (ISI vs. LSI), and *p* < 0.001 (LSI vs. HSI)). The rate of patients with fluid retention around the gallbladder on MRI was significantly higher, in decreasing order, in the LSI (52.9%), ISI (31.9%), and HSI (16.5%) groups (*p* = 0.012 (HSI vs. ISI), *p* = 0.007 (ISI vs. LSI), and *p* < 0.001 (LSI vs. HSI)). The preoperative WBC levels were significantly higher in the LSI group than in the HSI and ISI groups (*p* = 0.588 (HSI vs. ISI), *p* = 0.007 (ISI vs. LSI), and *p* = 0.002 (LSI vs. HSI)). Preoperative CRP levels were significantly higher, in decreasing order, in the LSI (6.5 mg/dL (1.51–16.09)), ISI (0.63 mg/dL (0.13–6.28]), and HSI (0.49 mg/dL (0.10–1.41)) groups (*p* = 0.027 (HSI vs. ISI), *p* < 0.001 (ISI vs. LSI), and *p* < 0.001 (LSI vs. HSI)). The number of patients who had undergone surgery within 48 h of disease onset was 90 (87.4%) of 103, 89 (76.7%) of 116, and 64 (62.7%) of 102 in the HSI, ISI, and LSI groups, respectively (*p* = 0.054 (HSI vs. ISI), *p* = 0.054 (ISI vs. LSI), and *p* < 0.001 (LSI vs. HSI)). There were no significant differences in the experience profiles of the operators.

### 3.3. Outcomes in Each MRI Group

Overall, bailout procedures were performed in 79 (24.6%) of 321 patients. Of these, conversions of the initial laparoscopic approach to open surgery and laparoscopic subtotal cholecystectomy were detected in 37 (11.5%) and 42 (13.1%) of 321 patients. The median operating time was 114 min (interquartile range: 88–144 min) (Table 2). 

Table 2 shows the outcomes of the three groups. Bailout procedures were identified in 7 (6.8%) of 103 patients in the HSI group, 31 (26.7%) of 116 patients in the ISI group, and 41 (40.2%) of 102 patients in the LSI group (*p* < 0.001). During the pairwise comparison, there were significant differences between each pair of groups (*p* < 0.001 (HSI vs. ISI), *p* = 0.043 (ISI vs. LSI), and *p* < 0.001 (LSI vs. HSI)). Conversion to open surgery was identified in zero (0%) of 103 patients in the HSI group, 10 (8.6%) of 116 patients in the ISI group, and 27 (26.5%) of 102 patients in the LSI group (*p* = 0.002 (HSI vs. ISI), *p* = 0.001 (ISI vs. LSI), and *p* < 0.001 (LSI vs. HSI)). Laparoscopic subtotal cholecystectomy was detected in 7 (6.8%) of 103 patients in the HSI group, 21 (18.1%) of 116 patients in the ISI group, and 14 (13.7%) of 102 patients in the LSI group (*p* = 0.044 (HSI vs. ISI), *p* = 0.461 (ISI vs. LSI), and *p* = 0.225 (LSI vs. HSI)).

The median operating times were 95, 110, and 138 minutes in the HSI, ISI, and LSI groups, respectively (*p* < 0.001). In the pairwise comparisons, there was a significant difference between every pair of groups (*p* = 0.013 (HSI vs. ISI), *p* < 0.001 (ISI vs. LSI), and *p* < 0.001 (LSI vs. HSI)). 

The amount of blood loss was small in each group (when the amount of blood loss was minimal, blood loss was counted as ‘0 mL’). Biliary injury occurred in two LSI patients, with no significant difference between the three groups in this regard. The rate of incidence of accidental gallbladder injury was significantly higher in the LSI group than in the HSI and ISI groups (*p* = 0.415 (HSI vs. ISI), *p* < 0.001 (ISI vs. LSI), and *p* < 0.001 (LSI vs. HSI)). 

The rate of intraoperative findings of severe inflammation was significantly higher in the order of LSI (87.3%), ISI (65.5%), and LSI (38.8%) (*p* < 0.001 (HSI vs. ISI), *p* < 0.001 (ISI vs. LSI), and *p* < 0.001 (LSI vs. HSI)).

Additionally, the postoperative hospital stay was significantly longer in the LSI group than in the HSI and ISI groups (*p* = 0.080 (HSI vs. ISI), *p* = 0.007 (ISI vs. LSI), and *p* < 0.001 (LSI vs. HSI)), but the median values were similar. The rates of patients with overall complications and major complications were low in each group, and there were no significant differences between groups. Overall, only one patient in the ISI group was readmitted, and no patient underwent reoperation. 

### 3.4. Identification of Risk Factors for Bailout Procedures

The univariate analysis identified eight risk factors (*p* ≤ 0.1) for bailout procedures: Severe inflammation findings on CT; the thickness of the gallbladder wall on MRI (≥8 mm); fluid retention around the gallbladder on MRI; the signal intensity of the gallbladder wall on MRI; the CRP level (≥8.0 mg/dL); the albumin level (≤3.8 mg/dL); Tokyo Guidelines severity grade II or III; and the time between onset and surgery (>24 h). In the multivariate analysis, ISI and LSI of the gallbladder wall on MRI (ISI vs. HSI, *p* = 0.004, odds ratio (OR) 3.71, 95% CI 1.51–9.10, and LSI vs. HSI, *p* < 0.001, OR 5.30, 95% CI 2.11–13.30) and the time between onset and surgery (>24 h) (*p* = 0.022, OR 2.19, 95% CI 1.12–4.29) were found to be independent risk factors for bailout procedures (Table 3). The final model showed a C-index of 0.77 (95% CI 0.71–0.82).

### 3.5. Identification of Risk Factors for Prolonged Operation

Based on the data collected from the overall study population, a prolonged operating time was defined as 144 min or longer. During the univariate analysis, eight risk factors (*p* < 0.1) for a prolonged operating time (≥44 min) were detected: Severe inflammation findings on CT; thickness of the gallbladder wall on MRI (≥8 mm); fluid retention around the gallbladder on MRI; the signal intensity of the gallbladder wall on MRI; the CRP level (≥8.0 mg/dL); the albumin level (≤3.8 mg/dL); Tokyo Guidelines severity grade II or III; and operator experience (≤4 years). In the multivariate analysis, LSI of the gallbladder wall on MRI (LSI vs. HSI, *p* < 0.001, OR 6.10, 95% CI 2.74–13.60) and operator experience (≤4 years) (*p* = 0.022, OR 2.03, 95% CI 1.11–3.71) were identified as independent risk factors for a prolonged operating time (≥144 minutes) (Table 4). The final model showed a C-index of 0.75 (95% CI 0.69–0.81). 

## 4. Discussion

This single-center retrospective study has presented two major novel findings for the surgical management of AC. First, LSI progression of the gallbladder wall in AC was significantly associated with a higher rate of bailout procedures, such as open conversion and laparoscopic subtotal cholecystectomy, and prolonged operating times. Second, the LSI of the gallbladder wall on MRI was the only independent risk factor for both bailout procedures and a prolonged operating time. Our results indicate that preoperative assessment of the signal intensity of the gallbladder wall on MRI is a novel and useful predictor for surgical difficulty during LC for AC. The routine assessment of MRCP and HASTE MRI at the time of AC diagnosis could provide surgeons with useful information for performing a safe surgery and provide appropriate surgical management in a limited time before subsequent early LC.

The overall open conversion rate was 11.5%, and the median operating time was 114 min. These findings were similar to those in previous studies in which early LCs were performed for AC [32,33,34,35]. As we hypothesized, LSI broadening in the gallbladder wall on preoperative MRI was significantly associated with a higher bailout procedure rate and longer operating time in early LC for AC. These results can be explained by the results of our previous study, which showed that the rate of inflammatory pathological changes such as necrosis, abscess formation, and fibrosis of the gallbladder wall significantly increased as LSI in the gallbladder wall broadened (27.4%, 84.8%, and 97.1% in the HSI, ISI, and LSI groups, respectively) [12]. There was a significant difference in operative outcomes between the HSI and LSI groups (bailout procedure rate: 6.8% vs. 40.2%; open conversion rate: 0% vs. 26.5%; operating time: 95 min vs. 138 min). Considering that less operator experience was an independent risk factor for prolonged surgery, it is recommended that a well-experienced surgeon conduct the operation for AC with LSI on MRI.

Previous studies have identified numerous risk factors for difficult LC, such as an advanced age, a male sex, the BMI, the American Society of Anesthesiologists physical status score, diabetes, previous abdominal surgery, gallbladder wall thickening, incarcerated stones in the gallbladder neck, fluid retention around the gallbladder, the body temperature, elevated WBCs, the CRP level, a low albumin level, a high bilirubin level, Tokyo Guidelines severity grade II/III AC, and elapsed time before surgery [17,18,19,20,21,22,23,24,25,26,27]. However, broadened LSI of the gallbladder wall on MRI was the only independent risk factor for both the bailout procedure and a prolonged operating time of LC for AC in the present study. We also assessed severe inflammation findings on CT, but they were not independent risk factors in the multivariate analysis. These results indicate that the assessment with MRCP better predicts difficult LC than preoperative CT or other previously reported risk factors.

MRCP detected CBDS in 16% of patients diagnosed with AC, which affected their treatment strategy. MRCP/MRI was performed in more than 90% of patients who underwent early cholecystectomy for AC, and PHD anatomies were confirmed in as many as 98% of these patients in the present study. These results were similar to those of previous studies [7,8,9,10]. Preoperative MRCP is a standard method for assessing the biliary anatomy before LC, not only for adult patients, but also pediatric patients who more often have anatomical variations [36]. The Tokyo Guidelines recommend that most patients with acute cholecystitis have early LC and that early LC should be performed before necrosis and fibrosis progress over time and the surgical difficulty increases [2,37]. After diagnosing AC, surgeons need to assess the surgical risk of subsequent LC, but often have limited time for examinations, especially in emergencies. Our results show that MRI is useful not only for assessing the presence of CBDS and biliary anatomy, but also for predicting the surgical difficulty due to inflammation. Although MRI is more expensive than other examinations, such as abdominal ultrasonography [37], it provides much information at once. Our MRI assessment method is available using the HASTE sequence captured during routine MRCP, as some studies have already reported [11,12,38,39], and can be quickly introduced as part of daily radiological studies. In conclusion, MRCP/MRI could be a useful option for the comprehensive surgical management of acute cholecystitis and decision making in emergencies.

Several limitations of the present study should be acknowledged. First, this was a retrospective and single-institution study. The criteria for bailout procedures varied among surgeons, and the operative time was largely dependent on the operator’s skills and experience. Additionally, some patients with a history of previous abdominal surgery or who had radiological findings that indicate severe inflammation of the gallbladder underwent planned open cholecystectomy based on the surgeon’s decision. Second, most of the patients diagnosed with AC more than 72 h after onset underwent delayed surgery following the 2013 Tokyo Guidelines [16]. Further validations such as prospective studies are needed regarding patients diagnosed as having AC more than 72 h after onset.

## Figures and Tables

**Table 1:** Characteristics of the three groups.

		Signal Intensity of the Gallbladder Wall on MRI			
	All (*n* = 321)	HSI (*n* = 103)	ISI (*n* = 116)	LSI (*n* = 102)	*p* Value
Age (years) *	62 (49–72)	60 (46–71)	62 (50–72)	64 (56–75)	0.061
Sex					
Male	214 (66.7)	60 (58.3)	84 (72.4)	70 (68.6)	0.079
Female	107 (33.3)	43 (41.7)	32 (27.6)	32 (31.4)	
BMI (kg/m^2^) *	24.7 (22.4–27.3)	24.9 (22.5–28.1)	24.6 (22.3–27.6)	24.6 (22.5–26.8)	0.953
ASA physical status					
I	106 (33.0)	39 (37.9)	36 (31.0)	31 (30.4)	0.953
II	192 (59.8)	60 (58.3)	70 (60.3)	62 (60.8)	
III	23 (7.2)	4 (3.9)	10 (9.8)	9 (8.8)	
Diabetes mellitus	54 (16.8)	17 (16.5)	19 (16.4)	18 (17.6)	0.964
Previous upper abdominal surgery	11 (3.4)	3 (2.9)	3 (2.6)	5 (4.9)	0.691
Body temperature *	36.9 (36.5–37.5)	36.8 (36.4–37.2)	36.9 (36.5–37.5)	37.1 (36.6–37.7)	0.014
Severe inflammation findings on CT	28 (8.7)	0 (0.0)	10 (8.6)	18 (17.6)	<0.001
Thickness of gallbladder wall on MRI (mm) *	7 (5–8)	6 (4–8)	7 (6–8)	7 (6–9)	<0.001
Incarcerated stones in the gallbladder neck on MRI	151 (47.0)	51 (49.5)	53 (45.7)	47 (46.1)	0.831
Fluid retention around the gallbladder on MRI	108 (33.6)	17 (16.5)	37 (31.9)	54 (52.9)	<0.001
WBC (/μL) *	12,140 (9500–15,630)	10,560 (8310–14,225)	11,130 (8817–14,995)	13,115 (10,760–16,100)	0.001
CRP (mg/dL) *	1.24 (0.18–7.6)	0.49 (0.10–1.41)	0.63 (0.13–6.28)	6.5 (1.51–16.09)	<0.001
AST (U/L) *	23 (18–32)	23 (18–31)	23 (18–32)	23 (19–38)	0.542
ALT (U/L) *	24 (16–40)	24 (16–38)	22 (16–41)	27 (17–46)	0.404
T-Bil (mg/dL) *	0.9 (0.6–1.5)	0.7 (0.6–1.1)	1.0 (0.6–1.6)	1.1 (0.8–1.7)	<0.001
Alb (g/dL) *	4.1 (3.8–4.4)	4.2 (4.0–4.4)	4.1 (3.8–4.4)	4.0 (3.6–4.2)	<0.001
Tokyo Guidelines Severity Grade					
I	250 (77.9)	93 (90.3)	93 (80.2)	64 (62.7)	<0.001
II	56 (17.4)	7 (6.8)	20 (17.2)	29 (28.4)	
III	9 (4.7)	3 (2.9)	3 (2.6)	9 (8.9)	
Time between onset of disease and surgery					
≤48 h	243 (75.7)	90 (87.4)	89 (76.7)	64 (62.7)	<0.001
>48 h	78 (24.3)	13 (12.6)	27 (23.3)	38 (37.3)	
Experience of the operator					
≤4 years	87 (27.1)	28 (27.2)	32 (27.6)	27 (26.5)	0.987
≥5 years	234 (72.9)	75 (72.8)	84 (72.4)	75 (73.5)	

Values in parentheses are percentages, unless indicated otherwise. * Values are medians (range). HSI, high signal intensity; ISI, intermediate signal intensity; LSI, low signal intensity. BMI, body mass index; ASA, American Society of Anesthesiologists; CT, computed tomography; MRI, magnetic resonance imaging; WBC, white blood cell; CRP, C-reactive protein; AST, aspartate aminotransferase; ALT, alanine aminotransferase; T-Bil, total bilirubin; Alb, albumin.

**Table 2:** Surgical outcomes of the three groups.

		Signal Intensity of the Gallbladder Wall on MRI			
	ALL (*n* = 321)	HSI (*n* = 103)	ISI (*n* = 116)	LSI (*n* = 102)	*p* Value
Surgical outcomes					
Bailout procedures	79 (24.6)	7 (6.8)	31 (26.7)	41 (40.2)	<0.001 ^†^
Conversion to open surgery	37 (11.5)	0 (0.0)	10 (8.6)	27 (26.5)	<0.001 ^‡^
Laparoscopic subtotal cholecystectomy	42 (13.1)	7 (6.8)	21 (18.1)	14 (13.7)	0.041 ^§^
Operating time (minutes) *	114 (88–144)	95 (80–117)	110 (81–132)	138 (116–171)	<0.001 ^||^
Blood loss (mL) *	0 (0–50)	0 (0–0)	0 (0–50)	23 (0–221)	<0.001 ^¶^
Biliary injury	2 (0.6)	0 (0.0)	0 (0.0)	2 (2.0)	0.100
Accidental gallbladder injury	195 (60.7)	52 (50.5)	66 (56.9)	77 (75.5)	<0.001 ^#^
Intraoperative findings					
Degree of inflammation assessed by the surgeon					
Mild to moderate	116 (36.1)	63 (61.2)	40 (34.5)	13 (12.7)	<0.001 **
Severe	205 (63.9)	40 (38.8)	76 (65.5)	89 (87.3)	
Postoperative outcomes					
Overall complications	18 (5.6)	4 (3.9)	5 (4.3)	9 (8.8)	0.259
Major complications	9 (2.8)	1 (1.0)	4 (3.4)	4 (3.9)	0.441
Reoperation	0 (0.0)	0 (0.0)	0 (0.0)	0 (0.0)	-
Postoperative hospital stay *	3 (3–4)	3 (3–3)	3 (3–4)	3 (3–6)	<0.001 ^††^
Readmission	1 (0.3)	0 (0.0)	1 (0.9)	0 (0.0)	1.000

LC, laparoscopic cholecystectomy; HSI, high signal intensity; ISI, intermediate signal intensity; LSI, low signal intensity. Values in parentheses are percentages, unless indicated otherwise. * Values are medians (range). ^†^ In pairwise comparisons, *p* < 0.001 (HSI vs. ISI), *p* = 0.043 (ISI vs. LSI), and *p* < 0.001 (LSI vs. HSI). ^‡^ In pairwise comparisons, *p* = 0.002 (HSI vs. ISI), *p* = 0.001 (ISI vs. LSI), and *p* < 0.001 (LSI vs. HSI). ^§^ In pairwise comparisons, *p* = 0.044 (HSI vs. ISI), *p* = 0.461 (ISI vs. LSI), and *p* = 0.225 (LSI vs. HSI). ^||^ In pairwise comparisons, *p*= 0.013 (HSI vs. ISI), *p* < 0.001 (ISI vs. LSI), and *p* < 0.001 (LSI vs. HSI). ^¶^ In pairwise comparisons, *p* = 0.001 (HSI vs. ISI), *p* = 0.001 (ISI vs. LSI), and *p* < 0.001 (LSI vs. HSI). ^#^ In pairwise comparisons, *p* = 0.415 (HSI vs. ISI), *p* = 0.009 (ISI vs. LSI), and *p* < 0.001 (LSI vs. HSI). ** In pairwise comparisons, *p* < 0.001 (HSI vs. ISI), *p* < 0.001 (ISI vs. LSI), and *p* < 0.001 (LSI vs. HSI). ^††^ In pairwise comparisons, *p* = 0.080 (HSI vs. ISI), *p* = 0.007 (ISI vs. LSI), and *p* < 0.001 (LSI vs. HSI).

**Table 3:** Univariate and multivariate analyses of risk factors for bailout procedures.

		Number (%) with Bailout Procedures	Univariate Analysis	Multivariate Analysis	
Variable	Category		*p* Value	OR (95% CI)	*p* Value
Age (years)	≥72	24/81 (29.6)	0.235		
	<72	55/240 (22.9)			
Sex	Male	57/214 (26.6)	0.272		
	Female	22/107 (20.6)			
BMI (kg/m^2^)	≥30	10/40 (25.0)	1.000		
	<30	69/281 (24.6)			
ASA physical status	≥III	3/23 (13.0)	0.218		
	≤II	76/298 (25.5			
Diabetes mellitus	Yes	10/54 (18.5)	0.301		
	No	69/267 (25.8)			
Past acute cholecystitis	Yes	6/19 (31.6)	0.425		
	No	73/302 (24.2)			
Previous upper abdominal surgery	Yes	3/11 (27.3)	0.735		
	No	76/310 (24.5)			
Body temperature (°C)	≥37.5	23/83 (27.7)	0.461		
	<37.5	56/238 (23.5)			
Severe inflammation findings on CT *	Yes	15/28 (53.6)	0.001	1.92 (0.66–5.52)	0.233
	No	64/293 (21.8)			
Thickness of the gallbladder wall on MRI (mm)	≥8	43/121 (35.5)	<0.001	1.87 (1.07–3.29)	0.029
	<8	36/200 (18.0)			
Incarcerated stones in the gallbladder neck on MRI	Yes	37/151 (24.5)	1.000		
	No	24/170 (23.5)			
Fluid retention around the gallbladder on MRI	Yes	38/108 (35.2)	0.002	1.24 (0.67–2.28)	0.480
	No	41/213 (19.2)			
Signal intensity of the gallbladder wall on MRI	HSI	7/103 (6.8)	<0.001	1	
	ISI	31/116 (26.7)		3.71 (1.51–9.10)	0.004
	LSI	41/102 (40.2)		5.30 (2.11–13.3)	<0.001
WBC (/μL)	≥15,000	28/95 (29.5)	0.203		
	<15,000	51/226 (22.6)			
CRP (mg/dL)	≥8.0	22/78 (28.2)	<0.001	1.18 (0.58–2.40)	0.641
	<8.0	15/243 (6.2)			
T-Bil (mg/dL)	≥1.5	26/88 (29.5)	0.245		
	<1.5	53/233 (23.8)			
Alb (g/dL)	≤3.8	31/90 (34.4)	0.014	1.21 (0.65–2.25)	0.557
	>3.8	48/231 (20.8)			
Tokyo Guidelines Severity Grade	II, III	28/71 (39.4)	0.002	0.985 (0.43–2.28)	0.972
	I	51/250 (20.4)			
Time between onset and surgery (hours)	>24	64/205 (31.2)	<0.001	2.19 (1.12–4.29)	0.022
	≤24	15/116 (12.9)			
Experience of the operator (years)	≤4	22/87 (25.3)	0.885		
	≥ 5	57/234 (24.4)			

OR, odds ratio; BMI, body mass index; ASA, American Society of Anesthesiologists; CT, computed tomography; MRI, magnetic resonance imaging; WBC, white blood cell; HSI, high signal intensity; ISI, intermediate signal intensity; LSI, low signal intensity; CRP, C-reactive protein; T-Bil, total bilirubin; Alb, albumin. * irregular thickening of the gallbladder wall, poor contrast enhancement of the gallbladder wall, increased density of fatty tissue around the gallbladder, membranous structures within the lumen, or perigallbladder abscess.

**Table 4:** Univariate and multivariate analyses of risk factors for prolonged surgery.

		Number (%) with Prolonged Surgery	Univariate Analysis	Multivariate Analysis	
Variable	Category		*p* Value	OR (95% CI)	*p* Value
Age (years)	≥72	22/81 (27.2)	0.659		
	<72	59/240 (24.6)			
Sex	Male	57/214 (26.6)	0.496		
	Female	24/107 (22.4)			
BMI (kg/m^2^)	≥30	9/40 (22.5)	0.846		
	<30	72/281 (25.6)			
ASA physical status	≥III	6/23 (26.1)	1.000		
	≤II	75/298 (25.2)			
Diabetes mellitus	Yes	9/54 (16.7)	0.125		
	No	72/267 (27.0)			
Past acute cholecystitis	Yes	3/19 (15.8)	0.422		
	No	78/302 (25.8)			
Previous upper abdominal surgery	Yes	1/11 (9.1)	0.302		
	No	80/310 (25.8)			
Body temperature (°C)	≥37.5	27/88 (30.7)	0.195		
	<37.5	54/233 (23.2)			
Severe inflammation findings on CT *	Yes	12/28 (42.9)	0.038	0.90 (0.31–2.65)	0.854
	No	69/293 (23.5)			
Thickness of the gallbladder wall on MRI (mm)	≥8	40/121 (33.1)	0.017	1.64 (0.93–2.89)	0.087
	<8	41/200 (20.5)			
Incarcerated stones in the gallbladder neck on MRI	Yes	36/151 (23.8)	0.609		
	No	45/170 (26.5)			
Fluid retention around the gallbladder on MRI	Yes	37/108 (34.3)	0.010	1.17 (0.64–2.15)	0.612
	No	44/214 (20.7)			
Signal intensity of the gallbladder wall on MRI	HSI	11/103 (10.7)	<0.001	1	
	ISI	22/116 (19.0)		1.72 (0.77–3.85)	0.184
	LSI	48/102 (47.1)		6.10 (2.74–13.60)	<0.001
WBC (/μL)	≥15,000	27/95 (28.4)	0.401		
	<15,000	54/226 (23.9)			
CRP (mg/dL)	≥8.0	29/78 (37.2)	0.007	1.08 (0.54–2.18)	0.828
	<8.0	52/243 (21.4)			
T-Bil (mg/dL)	≥1.5	22/88 (25.0)	1.000		
	<1.5	59/233 (25.3)			
Alb (g/dL)	≤3.8	29/90 (32.2)	0.086	1.05 (0.55–1.98)	0.888
	>3.8	52/231 (22.5)			
Tokyo Guidelines Severity Grade	II, III	27/71 (38.0)	0.008	1.31 (0.57–2.99)	0.514
	I	54/250 (21.6)			
Time between onset and surgery (hours)	>24	29/116 (25.0)	1.000		
	≤24	52/205 (25.4)			
Experience of the operator (years)	≤4	29/87 (33.3)	0.045	2.03 (1.11–3.71)	0.022
	≥5	52/234 (22.2)			

OR, odds ratio; BMI, body mass index; ASA, American Society of Anesthesiologists; CT, computed tomography; MRI, magnetic resonance imaging; WBC, white blood cell; HSI, high signal intensity; ISI, intermediate signal intensity; LSI, low signal intensity; CRP, C-reactive protein; T-Bil, total bilirubin; Alb, albumin. * irregular thickening of the gallbladder wall, poor contrast enhancement of the gallbladder wall, increased density of fatty tissue around the gallbladder, membranous structures within the lumen, or perigallbladder abscess.

## Data Availability

The data that support the findings of this study are available from the corresponding author (K.O.) upon reasonable request, due to restrictions of privacy.
